# Seizure Threshold, Suppression, and Expression as Dynamic Ontology in ECT

**DOI:** 10.1192/j.eurpsy.2026.11896

**Published:** 2026-07-31

**Authors:** G. J. Cowen, D. Nolasco, B. R. Carr

**Affiliations:** 1 School of Medicine, Wayne State University, Detroit, MI; 2 Department of Psychiatry and Behavioral Sciences, Stanford University, Stanford, CA; 3 Department of Psychiatry, University of Florida, Gainesville, FL, United States

## Abstract

**Introduction:**

Electroconvulsive therapy (ECT) discloses a paradox at the core of psychiatric intervention: its efficacy depends upon the induction of a seizure, yet the seizure is always already bounded by forces that both enable and constrain it. Stimulus technique, anesthetic mediation, cumulative exposure, and symptomatic change all act upon a shifting limit known as the seizure threshold. This threshold is not a fixed property but a dynamic frontier—rising across a treatment course, adapting with clinical response, and requiring continual recalibration.

**Objectives:**

To reconceive the seizure threshold as an ontological problem rather than a purely physiological measure: a moving boundary where suppression and expression, risk and efficacy, converge.

**Methods:**

Conceptual analysis, informed by psychiatric literature but oriented toward philosophy of science, interrogating how “threshold” functions less as a quantifiable marker than as a contingent interface that resists closure within static models.

**Results:**

The seizure threshold emerges as a dynamic limit shaped by multiple forces. It rises with treatment progression and often in parallel with symptomatic improvement, forcing clinicians to adjust stimulus dosing upward across a course. Anesthetic technique modulates its expression, altering seizure duration and cardiovascular tolerability, while stimulus parameters (unilateral vs. bilateral, pulse width, dosing algorithms) influence its trajectory. Clinical response itself feeds back into the system: as patients improve, the biological substrate of excitability shifts, altering the very boundary psychiatry relies upon. Failure to recalibrate risks underdosing and therapeutic non-response on the one hand, or excessive dosing and physiological burden on the other. What seems at first a technical issue is thus a conceptual one: the threshold is less a number to be measured than a liminal zone where psychiatry and anesthesia, biology and technique, illness and recovery meet.

**Conclusions:**

ECT’s reliability is paradoxically grounded in ontological instability. The seizure threshold adapts with time, technique, and therapeutic effect, obliging psychiatry to recognize efficacy as relational and contingent rather than fixed. To think the threshold philosophically is to see that suppression and emergence are not opposites but co-constitutive: the therapeutic act is continually reconstituted at their border. For psychiatry, this has practical implications—thresholds must be anticipated as moving targets, not assumed as constants—and conceptual ones, challenging the discipline to acknowledge that its most effective intervention depends on unstable, shifting conditions of possibility.

**Disclosure of Interest:**

None Declared

